# A (Bite) Force to Be Reckoned With

**DOI:** 10.1002/ajpa.70144

**Published:** 2025-10-28

**Authors:** Myra F. Laird, Megan A. Holmes, Claire E. Terhune, Andrea B. Taylor

**Affiliations:** ^1^ Department of Basic and Translational Sciences University of Pennsylvania Philadelphia Pennsylvania USA; ^2^ Department of Family Medicine and Community Health Duke University School of Medicine Durham North Carolina USA; ^3^ Department of Anthropology University of Arkansas Fayetteville Arkansas USA; ^4^ Department of Foundational Biomedical Sciences Touro University California Vallejo California USA

**Keywords:** craniodental, muscle, primate

## Abstract

**Objectives:**

Bite force has received significant attention in biological anthropology, but maximum bite force estimates for a single primate species often span hundreds of newtons. In this synthesis, we discuss the definitions of maximum bite force, review and highlight the variability in methods used to assess bite force in primates, and compare bite force ranges in macaques to bracket maximum force estimates between physiological and mechanical maxima.

**Materials and Methods:**

Methods of estimating bite force in primates were gathered from the literature along with published estimates of maximum bite force for macaques (*Macaca* sp.).

**Results:**

Maximum bite force can be defined physiologically or mechanically, and methods of estimating bite force can be grouped as in vivo, muscle‐based, and craniodental within these two definitions. Physiological estimates occur under natural conditions modulated by sensorimotor feedback, whereas mechanical maximum bite forces ignore muscular and neural limitations. Published maximum bite forces for macaques at the molars vary from 127 N to 898 N, a 771 N range. Using a bracketing approach suggested here, we narrow the estimated bite force range at the incisors to 487–503 N and 503–898 N for the molars.

**Discussion:**

This synthesis emphasizes the need for comparisons between in vivo, muscle‐based, and craniodental bite force methods in living primates. We propose bracketing bite force estimates between physiological and mechanical maxima in order to provide more reliable bite force estimates and improve understanding of how bite force relates to primate functional morphology and feeding ecology.

## 1 Introduction

Among the many measures of feeding system performance (Bennett 1989; Laird, Ross, et al. 2023), bite force has received significant attention in biological anthropology because it is informative for craniodental adaptations, community structure, food webs, diet, phylogeny, dominance and social display behaviors, and serves as an indirect measure of fitness (e.g., Anderson et al. 2008; Demes and Creel 1988; Meers 2002; Rayfield 2004; Vizcaíno and De Iuliis 2003; Wainwright 1991). However, accurately measuring and comparing bite force in primates has proved challenging, with maximum bite force estimates even for a single primate species often spanning hundreds of newtons (e.g., Holmes and Taylor 2021; Laird, Kanno, et al. 2023). In this synthesis, we define maximum bite force, highlight variability in bite force measurements in primates using macaques as an example, and propose the use of comparative brackets for maximum force estimates (as recommended by Taylor et al. 2025). We suggest that by applying specific criteria to establish lower and upper bounds of maximum bite forces for a given taxon, this variability can be reduced, providing more reliable bite force estimates and an improved understanding of primate functional morphology and feeding ecology.

We begin by borrowing from Bock and von Walhert's (Bock and Von Wahlert 1965) classic presentations of ‘function’, ‘faculty’, and ‘biological role’ in order to contextualize bite force in primates. The function of the system in this case is to elevate the mandible, produce bite force, and bring the teeth into contact with a food item or another substrate, and faculty or performance is the combination of form and function, here defined as the capacity to bite. Biological role is defined as the interaction between form, function, and the environment, or the context in which the capacity to bite is used.

Primates bite in a variety of biological roles including feeding and food extraction, some defensive and aggressive behaviors, tool use (e.g., Anderson et al. 2008; Demes and Creel 1988; Meers 2002; Rayfield 2004; Vizcaíno and De Iuliis 2003; Wainwright 1991), and as a means of exploration (e.g., grooming or during teething in juveniles; Poirier and Smith 1974). In an ideal world, biological anthropologists would be able to record in vivo and compare the bite forces used in each of these biological roles with an animal's maximum capacity to produce bite force. This would allow us to test whether selection is targeting maximum or submaximal forces, forces in specific biological roles, and/or how these targets vary between sexes and taxa. Alas, current methodological constraints prevent bite forces from being captured in most of the biological roles engaged in by primates, and maximum bite force capacity is instead used as a common point of comparison within and across primate taxa.

Maximum bite force is broadly defined as the maximum force that can be produced on the dentition, and in biological anthropology, this definition typically spans both physiological maximum bite forces that occur under natural conditions modulated by sensorimotor feedback, and mechanical maximum bite forces that ignore muscular and neural limitations. All bite forces associated with biological roles are necessarily physiological, but not all physiologically generated bite forces can be meaningfully linked to biological roles. For example, vertical forces generated by marmosets during transducer biting were substantially higher than those used during simulated gouging in the laboratory, which was supported by low force estimates measured from wild gouges based on substrate material properties and mark sizes (Vinyard et al. 2009). Thus, the methods used to capture physiological maximum bite force outside of a natural environment must be analogous to biting in the wild to accurately capture a biological role (Vinyard et al. 2009). Mechanical maximum bite force reflects the maximum capacity of the animal to generate bite force, but these forces are unlikely to ever occur in a living animal because of sensorimotor feedback and protective mechanisms (e.g., Trulsson and Johansson 2002; Trulsson 2007). As a result, mechanical maximum bite forces should always be larger than physiological maximum bite forces, and this creates a theoretical upper and lower bound, respectively, with which to bracket maximum bite forces.

Comparisons of maximum bite force across primates typically focus on either maximum physiological or maximum mechanical estimates. In vivo estimates of bite force can fall under either the physiological or mechanical definitions of maximum bite force, but in vivo bite force data from extant primates are extremely limited and few physiological measures have been matched with a clear biological role (e.g., Chazeau et al. 2013; Dechow and Carlson 1990; Hylander 1979; Laird, Kanno, et al. 2023; Laird et al. 2024; Thomas et al. 2015; Vinyard et al. 2009; Zablocki Thomas et al. 2018). The majority of maximum bite force estimates in extant primates (and all estimates in fossils) derive from indirect methods based on muscular, skeletal, and/or dental anatomy, including finite‐element analysis (FEA) or multibody dynamics analysis (MDA; e.g., Antón 1990, 1994; Constantino et al. 2010; Curtis et al. 2008; Dechow and Carlson 1990; Demes and Creel 1988; Deutsch et al. 2020; Dumont et al. 2011; Eng et al. 2013; O'Connor et al. 2005; Perry et al. 2011; Spencer and Demes 1993; Smith et al. 2015; Strait et al. 2009). In mammals, muscle force is converted to bite force at a particular point on the occlusal surface, and this conversion depends upon a number of variables intrinsic to an individual, such as muscular, craniofacial, and dental morphology, as well as extrinsic factors such as the geometric and mechanical properties of a food item or substrate (Molnar and Gantt 1977; Olejniczak et al. 2008; Oyen and Tsay 1991; Schwartz 2000; Shellis et al. 1998; Smith et al. 2005). These indirect methods involve one or more of these intrinsic and/or extrinsic variables and can also fall under either the physiological or mechanical definitions of maximum bite force.

Here we contextualize the range of variation in published primate bite forces by comparing multiple indirect and in vivo methods for estimating bite force (Table 1). We then compare published bite force estimates for a single primate taxon, macaques, and offer an approach for bracketing bite force ranges. The goal in this bracketing is to create a range of maximum bite forces that span both the physiological and mechanical definitions in order to encompass multiple potential selection targets, facilitate comparisons between taxa, and clarify relationships between force estimates and feeding morphology and ecology.

**TABLE 1:** Summary of the methods used to calculate bite force in primates.

Method	Example primate citations	Direct or indirect^a^	Strengths	Limitations
*Muscle‐based*				
PCSA	Perry et al. 2011; Eng et al. 2013; Deutsch et al. 2020; Holmes and Taylor 2021, 2023	Indirect	Estimates maximum force capacity Can be measured in cadaveric specimens Related to dietary adaptations, social signaling, and competition	Assumes a perfect contraction with 100% fiber activation across a fiber's full length and rotation Fiber type and leverage data are required to convert PCSA values to bite force Unclear how PCSA values relate to in vivo bite forces
Finite element analyses (FEA)	Wroe et al. 2010; Strait et al. 2009	Indirect	Measures maximum force capacity, can be measured in cadaveric specimens, allows modeling of fossil or extant taxa without available muscle architecture	Limited application of submaximal biting or chewing (but see Panagiotopoulou et al. 2023)
*Skeletal and dental*				
Mechanical advantage	Demes and Creel 1988; O'Connor et al. 2005; Wroe et al. 2010; Strait et al. 2009	Indirect	Readily measurable in skeletal remains, including fossils	Requires muscle force estimate to convert to bite force
Food force to fracture	Lucas et al. 1994; Hill et al. 1995	Indirect	Force used during feeding	Force to fracture is potentially moderated by non‐oral behaviors Measures of force are recorded mechanically outside of the oral cavity Dependent upon accurately replicating food fracture Requires replication on many foods Maximum force values likely exceed food fracture values
Dental chipping	Constantino et al. 2010	Indirect	Applicable to fossils and extant primates Reflects in vivo behaviors	Captures bite forces used in a variety of roles
Dental histology	Chai 2020	Indirect	Reflects the maximum force the teeth can withstand	Destructive
In vivo				
Muscle stimulation	Dechow and Carlson 1990	Direct	Maximally contracts the stimulated muscle Can include many muscles Doesn't depend on the animal's willingness to bite Should theoretically record forces near the animal's maximum	Doesn't account for working and balancing side differences Requires stimulating at the geometry of the electrode and regulating the amount of current Challenging to include all muscles, such as medial pterygoid Fatigue and reflex circuits may affect force output Invasive procedures
Transducer	Laird, Kanno, et al. 2023; Laird et al. 2024	Direct	Non‐invasive procedures Inclusive of all in vivo muscles and recruitment patterns	Depends on the animal's willingness to bite Unlikely to record an animal's maximum bite force Requires behavioral training

^a^ Direct methods are defined as those that record force (N) from the oral cavity in a living primate, whereas indirect methods of assessing bite force are measured outside of the oral cavity in a living animal.

## 2 In Vivo Methods

In vivo bite force estimates measured from living animals are paramount for contextualizing expected variation in bite force estimates between methods. The largest in vivo bite forces are thought to occur during aggression or defense, and larger in vivo forces have been noted in sexes and species that more often engage in aggressive tactics/behaviors, including mouse lemurs (*Microcebus murinus*; Chazeau et al. 2013; Dumont and Herrel 2003; Herrel et al. 2009; Thomas et al. 2015). By contrast, the maximum bite forces used during chewing, exploration, and tool use are expected to be smaller, with larger forces used during isometric biting on hard objects. In primates, in vivo bite forces have been recorded through both involuntary muscle stimulation and voluntary biting using a force transducer (a device where primates bite on metal plates that pull on an isometric load cell registering force; Herrel et al. 2001).

Work by Dechow and Carlson (1990) involved measuring unilateral bite forces using a force transducer in macaques by electrically stimulating the masseter muscles. These data have subsequently been used as a benchmark for bite force estimates and FEA models (e.g., Eng et al. 2013; Kupczik et al. 2007; Vinyard et al. 2008; Wang et al. 2012). However, in normal physiological conditions, in vivo bite force is under neurophysiological control, meaning that sensorimotor feedback within the muscle and nervous system modulates the total amount of force an individual can voluntarily produce. The application of bite force to a food item, the occlusal surface, or other substrate is modulated by sensory feedback from the jaws, teeth, skin, and mucosa that reflects the size, shape, and stability of the substrate and safety of the feeding system. In particular, the mammalian periodontal sensory afferents communicate information on the magnitude, orientation, and location of bite force being applied at the teeth to the brain and masticatory muscles (Byers 1985; Hannam 1969; Hannam and Farnsworth 1977; Johnsen and Trulsson 2003, 2005; Larson et al. 1981; Loescher and Robinson 1989; McIntosh et al. 2002; Ross et al. 2007). Importantly, electrical stimulation artificially activates the jaw muscles, overriding these neurophysiological controls on voluntary bite force that reduce the risk of injury (Lund 1991; Paphangkorakit and Osborn 1997, 1998; Sessle 2006; Trulsson 2007; Türker 2002; Turker et al. 2007; Vinyard et al. 2008). As such, this approach falls under the mechanical definition of maximum bite force and should result in forces higher than those used during normal activities. We note that at the incisors, stimulated tetanic bite force values from Dechow and Carlson (1990) for *Macaca mulatta* are smaller than voluntary transducer values reported by Laird (this manuscript), and *Macaca mulatta* stimulated tetanic bite forces at the molars were similar to voluntary transducer values for *Macaca fascicularis* (Hylander 1979). This difference likely reflects the fact that the 1990 stimulations were performed unilaterally in just the masseter muscle, as well as the larger body size of *M. mulatta* compared to *M*. *fascicularis*. Bite forces from involuntary approaches are highly dependent on the level of stimulation, access to the muscle(s), and the muscle's innervation.

Voluntary bite force recorded using a force transducer has been well validated in other animals and can capture bite force in a range of behaviors from aggression to exploration (e.g., Aguirre et al. 2003; Herrel et al. 2001, 2004, 2005, 2008, 2009; Verwaijen et al. 2002). Maximum voluntary bite forces are defined as physiological. However, voluntary bite forces are only available for a few nonhuman primates—*Macaca* (Hylander 1979; Laird, unpublished), *Galago* (Hylander 1977, 1979), *Mi. murinus* (Chazeau et al. 2013; Thomas et al. 2015; Zablocki Thomas et al. 2018), *Hapalemur* (Vinyard et al. 2008); *Callithrix* (Vinyard et al. 2009), and recently *Sapajus*, *Eulemur*, and *Varecia* (Laird, Kanno, et al. 2023; Laird et al. 2024). Only two studies have compared voluntary bite force across multiple species of primates (Vinyard et al. 2019; Laird et al. 2024), and only data from *Callithrix*, *Hapalemur*, *Mi. murinus*, and *Sapajus* have been aligned with their biological roles of gouging and feeding (Vinyard et al. 2008, 2009; Zablocki Thomas et al. 2018; Laird, Kanno, et al. 2023). Voluntary bite force is dependent on the animal's motivation to bite, and these recorded forces are likely to be lower than those collected using involuntary stimulation. Most analyses of voluntary bite force include only the highest recorded value(s), but smaller forces collected during voluntary biting may be informative to the range of in vivo bite forces used in different behaviors.

## 3 Indirect Estimates of Bite Force in Primates: Muscle‐Based Methods

Muscle physiological cross‐sectional area (PCSA) is often used to estimate jaw adductor muscle force, which then is converted to bite force. Physiological cross‐sectional area is an estimate of maximum force capacity of the jaw adductor muscles measured ex vivo, a mechanical maximum bite force, but this force capacity is unlikely to be produced in living animals because of sensorimotor feedback and protective mechanisms (e.g., Trulsson and Johansson 2002; Trulsson 2007). Therefore, all else being equal, PCSA‐derived muscle and bite force estimates are expected to be higher than those collected in vivo because of this assumed ‘maximal’ contraction. With this in mind, variation in primate jaw adductor PCSA values and their derived bite force estimates have been functionally and adaptively related to diet, social signaling, and aggressive biting during competition, and employed in intra‐ and interspecific scaling analyses of the feeding system (for a recent comprehensive review of primate jaw‐muscle architecture, fiber‐type phenotype, and jaw‐muscle performance see Taylor et al. 2025).

Bite force estimates based on muscle architecture (i.e., PCSA) were found to be similar to voluntary in vivo bite forces recorded during aggressive behaviors using a force transducer in bats, rodents, ants, and grasshoppers (Ginot and Blanke 2024; Ginot et al. 2018; Herrel et al. 2008; Püffel et al. 2023). However, there are no such published comparisons for primates. More recently, diffusible iodine‐based contrast‐enhanced computed tomography (diceCT) has been used to estimate PCSA, although there are differences between diceCT and dissection‐based methods depending on the muscle and taxa (Dickinson et al. 2018, 2019, 2020; Santana 2018; Sullivan et al. 2019). In sum, these muscle‐based bite force estimates offer a robust platform for comparing mechanical maximum force measures of the jaw adductors among taxa.

Muscle architecture data have also been used to estimate bite force in primates using simulated models such as FEA, MDA, and other Hill‐type muscle models (e.g., Curtis et al. 2008; Dumont et al. 2011; Fitton et al. 2012; Iriarte‐Diaz et al. 2017; Laird et al. 2024; Ledogar et al. 2018; Shi et al. 2012; Smith et al. 2015; Strait et al. 2009). These approaches use muscle architecture data to estimate bite force within an animal's musculoskeletal geometry. Like other muscle‐based estimates of bite force, these simulated models are based ex vivo on the maximum force capacity of a muscle, but modeling can occur at either maximum or submaximal levels. As such, bite forces from simulated muscle models can be classified as either mechanical or physiological maximum bite forces. Curtis et al. (2010) compared bite force estimated using MDA with estimates from in vivo experiments in *Sphenodon* and found that modeled bite forces were substantially lower than experimental values. These authors note that the mismatch may result from the limitations of the model in capturing complex muscle architecture that influences muscle force production (Curtis et al. 2010). By contrast, FEA models used to predict maximum bite force in rats, guinea pigs, and squirrels corresponded well with transducer in vivo forces (Cox et al. 2012).

For primates, a macaque FEA model was found to produce maximum bite forces similar to those obtained from in vivo muscle stimulations (Ross et al. 2005), and Hill‐type muscle simulation models using muscle architecture were found to accurately simulate transducer in vivo bite forces in *Eulemur* and *Varecia* (Laird et al. 2024). Collectively, these studies suggest methods such as FEA and muscle modeling have the potential to accurately simulate in vivo biting, but additional studies are needed to relate these approaches, as well as studies comparing biting using static vs. dynamic models and comparisons between modeling techniques (e.g., Panagiotopoulou et al. 2023), particularly within a single individual.

## 4 Indirect Estimates of Bite Force in Primates: Skeletal and Dental Methods

Indirect estimates of bite force in fossil primates are typically based on variation in extant primate craniodental morphology. Among the most common is the assessment of an intrinsic determinant of bite force, mechanical advantage (MA), which is defined as the ratio of each muscle's lever arm (i.e., the perpendicular distance from the mandibular axis of rotation to the muscle's line of action) to the load arm (i.e., the distance from the axis of rotation to the bite point). Mechanical advantage determines the efficiency of converting muscle force to bite force (Greaves 1978, Greaves 1983, Greaves 2012; Radinsky 1981; Spencer 1999) and can be readily estimated from skeletal remains. Critically, translating MA into an approximation of in vivo bite force requires an estimate of the muscle force being converted to bite force, and these estimates are typically of maximum capacity, making bite force estimates using MA mechanical. Demes and Creel (1988) used MA multiplied by skeletal estimates of contractile force for the temporalis and masseter muscles, which they estimated respectively via the cross‐sectional area of the infratemporal fossa and masseter origin length in extant primates and fossil hominins. Expanding upon Demes and Creel (1988), O'Connor et al. (2005) used skeletal cross‐sectional areas with a correction factor for each muscle derived by comparing skeletal measurements and PCSA from a human sample (from Weijs and Hillen 1985, 1986), and Wroe et al. (2010) used FEA and corrected cross‐sectional areas from O'Connor et al. (2005). Eng et al. (2013) further expanded on these approaches by including species‐specific estimates of muscle architecture. These analyses used slightly different methods of estimating muscle force for use in MA, but they offer an approach for indirect bite force estimates in fossil primates, including fossil hominins, for which soft tissues are not available.

Another indirect method of measuring bite force in primates is based on the force required to fracture food items. Orangutans (*Pongo pygmaeus*), for example, were observed fracturing two types of seeds with differing material properties. The seeds were then loaded using a universal tester outfitted with metal plates and metal casts of orangutan teeth to mimic the fracture pattern and record the force needed for fracture (Lucas et al. 1994). A similar approach was used to assess bite force in *Macaca fuscata* through the fracture of woody plant galls (Hill et al. 1995). These food fracture methods capture the bite force used during feeding/food extraction and provide strong evidence of high‐force use—food fracture values readily exceeded those measured on a force transducer in tufted capuchins (Laird, Kanno, et al. 2023). This indirect method of estimating maximum bite force is physiological and has a clear biological role related to feeding and food access. However, food fracture approaches to estimating maximum bite force must be accurately replicated in a laboratory and may omit variation resulting from food selection.

Other indirect methods of estimating bite force in extant and fossil primates use the dentition to analyze the force required to produce enamel chips near the occlusal edge (Constantino et al. 2010), and as a function of an enamel fracture constant and measures of enamel thickness and dentin horn angle from dental histological sections using a finite element stress analysis (Chai 2020). Bite force estimates from dental histological sections likely represent the maximum force the teeth can withstand without fracture, a mechanical definition, but it is unclear whether enamel chipping reflects a mechanical or physiological maximum bite force. Certainly, enamel chipping occurs through physiologically generated forces, although the biological role is not clear as enamel chips can result from a variety of behaviors, but calculations of force from enamel chips reflect the failure threshold of the enamel tissue, suggesting a mechanical maximum. At present, we do not classify this method as either mechanical or physiological and exclude it from consideration in bracketing (Table 2).

**TABLE 2:** Published estimates of maximum incisal and molar muscle and bite force for macaques. Muscle or bite force estimates are given as a range, average, and/or maximum reported depending on the publication. M = male, F = female, FEA = finite element analysis, MDA = multibody dynamics analysis.

Muscle or bite force (in newtons, N)	Location	Category (Figure 1): Method	Species (sample size)	References
Range: 92.18–101.99 N	Incisors	Physiological: In vivo voluntary: transducer	*Macaca fascicularis* (*n* = 4: 3F adults, 1 M subadult)	Hylander (1979)
Maximum: 319.14 N	Incisors	Physiological: In vivo voluntary: transducer	*Macaca mulatta* (*n* = 4: 2 M adults, 2F adults)	Laird (unpublished)^a^
Mean range: 133.1–151.1 N	Incisors	Mechanical: In vivo involuntary: unilateral stimulation	*Macaca mulatta* (*n* = 96: 81F adults, 15 M adults)	Dechow and Carlson (1990)
Maximum: 395.55 N	Incisors	Mechanical: Muscle: PCSA + leverage	*Macaca silenus* (*n* = 1: 1 M adult)	Deutsch et al. (2020)
Maximum: 503.74 N	Incisors	Mechanical: Muscle: PCSA + leverage	*Macaca sylvanus* (*n* = 2: 1 M adult, 1F adult)	Deutsch et al. (2020)
Mean range: 390–500 N	Incisors	Mechanical: Muscle: PCSA + fiber‐type phenotype	*Macaca fascicularis* (*n* = 6: 1 M adult, 3F adults, 2 M subadults)^b^	Holmes and Taylor (2023)
Maximum: 487 N	Incisors	Physiological: Craniodental: bite marks, food fracture microhardness	*Macaca fuscata* (NA) ^c^	Hill et al. (1995)
Mean range: 286.2–369.3 N	M1–M3	Mechanical: In vivo involuntary: unilateral stimulation	*Macaca mulatta* (*n* = 96: 81F adults, 15 M adults)	Dechow and Carlson (1990)
Average: 235 N; Maximum: 333 N	M1–M3	Physiological: In vivo voluntary: transducer	*Macaca fascicularis* (*n* = 4: 3F adults, 1 M subadult)	Hylander (1979)
Range: 898.42 ± 40.88 N (Male); 715 ± 48.6 N (female)	M2	Mechanical: Muscle: PCSA + leverage + skeletal cross‐sectional area	*Macaca fascicularis* (*n* = 2: 1F adult, 1 M adult)	Eng et al. (2013)
Maximum: 133.7 N	M2	Mechanical: Muscle: FEA and MDA	*Macaca fascicularis* (*n* = 1: 1 M adult)	Curtis et al. (2008)
Maximum: 290 N	M1–M3	Mechanical: Muscle: FEA	*Macaca fascicularis* (*n* = 1: 1 M adult)	Shi et al. (2012)
Maximum: 133.56 N	M1	Mechanical: Craniodental: skeletal leverage	*Macaca fascicularis* (*n* = 6: 5F adult, 1 M adult)	Lucas (2012)^c^
Maximum: 127.07 N	M1	Mechanical: Craniodental: skeletal leverage	*Macaca fuscata* (*n* = 2: 1F adult, 1 M adult)	Lucas (2012)^d^
Range: 624–875 N	M1–M3	Unknown: Craniodental: dental chipping	*Macaca* spp.^e^ (*n* = 5: 3F adults)	Constantino et al. (2010)

^a^ Unpublished *Ma. mulatta* bite forces were approved and collected under the University of Pennsylvania IACUC protocol 805870.

^b^ Not all specimens were used in estimates of muscle force and bite force.

^c^ Bite forces for *Ma. fuscata* were estimated from tooth marks on woody galls.

^d^ The largest force estimate from Lucas (2012) was reported on the M_3_ with a maximum of 143.7 N for *Ma. fascicularis* and 127.42 N for *Ma. fuscata*.

^e^ The Constantino et al. (2010) sample contained five adults: one *Macaca nemestrina* of unknown sex, one *Ma. sylvanus* of unknown sex, and three female *Ma. mulatta*.

We emphasize the need for future direct comparisons between maximum bite force estimates from in vivo, muscle‐based, and craniodental approaches within a single animal (Table 1). Methods that include muscle force capacity (e.g., FEA models) might be expected to yield bite force estimates similar to those obtained from muscle‐based approaches, but skeletal proxies of muscle cross‐sectional area almost certainly underestimate or overestimate (depending on the muscle) force capacity by excluding architectural variables that can impact differences in muscle force, such as fiber length and pinnation angle (e.g., Rockenfeller et al. 2024). Similarly, direct comparisons can help to classify enamel chipping approaches and differences between muscle‐based and in vivo estimates.

## 5 Comparing and Bracketing Bite Forces in Macaques

To contextualize the large range of variation in published bite forces for extant primates, we consider a commonly studied nonhuman primate—macaques. Published maximum bite force estimates for *Macaca* are derived from a variety of methods including muscle architecture and fiber‐type phenotype (Holmes and Taylor 2021), craniodental methods such as dental chipping and skeletal leverage (e.g., Constantino et al. 2010; Lucas 2012), and in vivo data (e.g., Dechow and Carlson 1990), and each approach carries the potential for method‐specific ranges of error and biases. To maximize the number of methods and range of variation, we include data from multiple species of macaques, although muscle (Antón 1999; Terhune et al. 2015) and, by extension, bite force, is expected to vary between species, sexes, and with body size in adult macaques. For each study, we indicate the location of bite force on the toothrow (here, incisors vs. molars) because all else being equal, bite force estimates at the molars are theoretically expected (Greaves 1978; Spencer 1998, 1999) and empirically demonstrated (e.g., Dechow and Carlson 1990) to exceed those at the incisors based on differences in jaw leverage.

**FIGURE 1:**
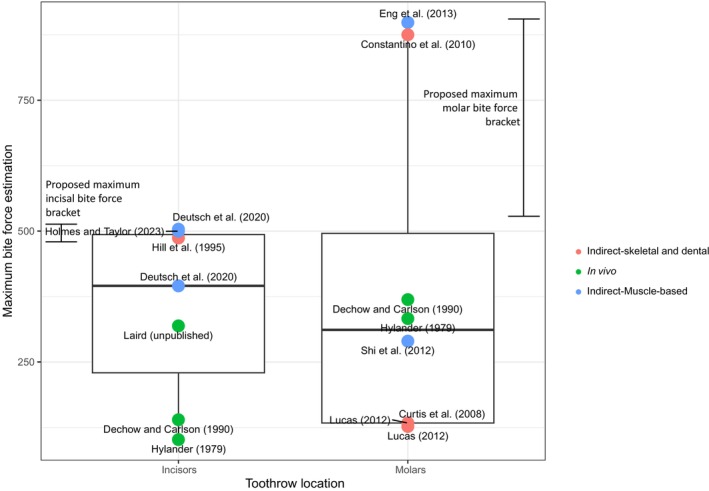
Published bite force estimates for macaques at the incisors and molars (see also Table 2). In vivo forces are those recorded from living primates, whereas indirect methods are ex vivo from skeletal or dental morphology or are muscle‐based. For publications reporting a range, only the maximum bite force values are plotted. Proposed bracketed ranges based on comparisons across methods were added for both the incisors and molars. These brackets assume that muscle‐based maximum bite force estimates should be higher than both stimulated and voluntary in vivo measures, and molar bite force estimates should be absolutely larger than those from the incisors.

Published macaque bite forces at the incisors are as low as 92.18 N (an in vivo physiological measure; Hylander 1979) and as high as 503.74 N (muscle‐based mechanical measure; Deutsch et al. 2020; range = 411.56 N) (Figure 1; Table 2). The upper bound value is consistent with another muscle‐based mechanical measure reported by Holmes and Taylor (2023; 500 N; Figure 1; Table 2). Both voluntary and involuntary in vivo transducer estimates are lower than those based on muscle architecture data. We also report a previously unpublished maximum voluntary bite force recorded on the incisors of an adult male *Ma. mulatta* of 319.14 N,^1^ which is substantially larger than previously reported voluntary and involuntary in vivo incisor bite forces (Hylander 1979; Dechow and Carlson 1990; Figure 1; Table 2).

The highest published molar bite force was estimated using a combination of muscle architecture and leverage data (898.42 N; Eng et al. 2013), followed closely by dental chipping (875 N; Constantino et al. 2010) (Figure 1; Table 2). The Eng et al. (2013) estimate is a mechanical definition of maximum bite force, whereas the dental chipping measure is likely physiological. As expected, bite force maxima based on skeletal and dental dimensions are larger at the molars compared with the incisors. Interestingly, some studies using skeletal leverage, FEA, and MDA estimated smaller macaque molar bite force values than those recorded using in vivo methods; some of these differences may relate to comparing between sexes and across species and a range of body sizes. The molar bite force range of ~771 N (127.07 N–898 N; Constantino et al. 2010; Eng et al. 2013; Figure 1; Table 2) is substantially larger than the incisor range. For context, 771 N approximates the amount of force produced on the ground from a 170 lb. (77.5 kg) person standing still. These values indicate that intraspecific/intrageneric variation in macaque in vivo bite force estimates may be large, as suggested by macaque muscle architecture data (Terhune et al. 2015).

The wide range in bite force values and mix of values derived under mechanical and physiological definitions makes it difficult to draw meaningful comparisons about adaptations to maximum bite force. We thus propose bracketing maximum bite force ranges between the physiological maximum bite force—as a minimum bound on the amount of force an animal can produce—and the mechanical maximum—the maximum force capacity if all force from perfect muscle contractions is fully translated to a bite point. We recognize the variability of these bounds, as many physiological measures depend on the motivation of the animal and mechanical measures are reliant upon the individual's health status. Nevertheless, these bounds provide a reasonable starting point for comparing maximum bite forces using minimum physiological and maximum mechanical theoretical thresholds. It follows that species‐specific estimates of mechanical maximum bite force that fall well below physiological measures for a particular location on the tooth row should potentially be reassessed, as should physiological estimates that are well above mechanical bite force estimates. We do not include dental chipping measures in these bounds as we currently lack information on how these measures fall within the physiological or mechanical definitions of maximum bite force, but this can be amended with such comparisons in the future.

Following these guidelines, we bracket the macaque maximum bite force estimates in Table 2:

Incisors: Using the largest reported physiological maximum force from the incisors—487 N (Hill et al. 1995)—becomes the lower bound of the range. The highest reported mechanical measure (~503 N; Deutsch et al. 2020) becomes the upper bound of the range. This yields a narrowed range of variation for maximum bite force on macaque incisors of 487–503 N, reducing the range from ~411 N to ~16 N (Figure 1).

Molars: The highest reported physiological bite force is 333 N (Hylander 1979) and the highest reported mechanical estimate of bite force is 898.4 N (Eng et al. 2013). However, molar bite forces are expected to be absolutely larger than those at the incisors based on jaw leverage (Greaves 1978, 1983, Greaves 2012; Radinsky 1981; Spencer 1999) and the in vivo maximum at the incisors is 503 N. Using this upper bound of the incisor in vivo range as the minimum in vivo bound for the molars, the molar range thus becomes 503–898 N (Table 2), reducing the range of variation in estimates for the molars from 771 N to ~395 N (Figure 1).

## 6 Discussion and Future Directions

Maximum bite force informs questions about morphology, behavior, and the environment in both extant and fossil primates. Despite numerous methods used to estimate bite force in primates, few studies have compared force estimates between approaches. We found that published maximum bite force estimates for macaques vary widely, by as much as 771 N, depending on method and toothrow location. We propose bracketing maximum bite force estimates between the reported physiological and mechanical maxima to narrow the range of force variation and facilitate comparisons between/among taxa.

While this type of bracketing is currently limited to the few extant primates with available mechanical and physiological bite force estimates, our macaque example provides a useful framework for bracketing the large range of bite force estimates currently in the literature for these taxa and for data collected on additional taxa in the future. We recognize that factors beyond differences in methods, such as age, sex dimorphism, population, species, and health status, account for some of the variation in force across studies. Male *Ma. fascicularis*, for example, have significantly larger masseter and temporalis PCSAs than conspecific females (Terhune et al. 2015), and in vivo bite forces recorded across human populations vary widely (e.g., Waltimo and Könönen 1993; Eng et al. 2013). Future work may tighten extant primates' ranges even further (e.g., controlling for species and sex), but the opportunistic nature of primate sampling suggests comparative bracketing will be a useful approach for many taxa.

As noted earlier, methods for estimating bite force in primates have been aimed largely at maximum bite force, but the target of selection may be submaximal forces. Physiological maximum bite forces are below maximum force capacity, but these maxima may not align with biological roles, as in the case of tree‐gouging common marmosets (Vinyard et al. 2009), assuming the laboratory environment adequately simulates the natural environment. We don't, however, know how closely PCSA‐derived bite force estimates approximate the recorded gouging forces. It is also likely that for certain behaviors, the production of large bite forces may be less important for the feeding system than other performance factors such as fatigue resistance (e.g., Ranieri and Di Lazzaro 2012). Further tests of changes in muscle mass and PCSA in relation to muscle fiber type will elucidate the role of selection for fatigue resistance in the primate jaw adductors. The target of selection will almost certainly vary between sexes, taxa, and with behavior, and critical evaluation of existing and future bite force data in the context of a given question/hypothesis will ultimately improve estimations of bite force ranges and modeling of bite force in both extant and fossil taxa.

*What do we do with an extant primate or fossil with limited or no bite force data?*

Physiological estimates are currently not available for most extant primates and cannot be obtained for any fossil taxa. Thus, the only option for bite force estimates in many extant and all fossil taxa is craniodental remains, although published PCSA data are available for the full complement of jaw adductors for nearly 40 extant primate species (Taylor et al. 2025). In such cases, estimates of maximum force capacity must rely on a combination of indirect methods. We intentionally do not categorize some methods as ‘better’ or ‘worse’ than others. Rather, we advocate for the consilience of as many methods as possible and for common sense comparisons between approaches that reconsider mismatched estimates. With limited or no physiological data, where feasible, we suggest using at least two different indirect methods to estimate bite force—preferably from different methodological categories. In living primates, this could be a combination of muscular and food fracture estimates, as one example. Bite force estimates for fossils could be modeled using FEA in addition to MA and/or dental chipping, as examples.

Importantly, all estimates discussed here assess vertical bite force. Primate mastication, and likely other uses of bite force, consist of vertically, laterally, and anteroposteriorly directed force components, but at present, non‐vertical forces are not measurable in vivo using a transducer. Triaxial gages measuring strain on the mandible and patterns of jaw adductor muscle activation suggest significant variation in the magnitude, directionality, and timing of bite forces during feeding (Hylander 1977; Hylander et al. 1998, 2000; Ravosa 1988, 1990; Ram and Ross 2018). Aggressive and defensive biting presumably favor large vertical bite forces with minimal laterally and anteroposteriorly directed forces; emphasizing vertical bite forces in such contexts would be critical to stabilize and avoid injury at the temporomandibular joint during these activities (DuBrul 1974; Maynard Smith and Savage 1959; Noble 1973; Spencer 1999). By contrast, laterally directed jaw movements have been related to aspects of the oral environment during mastication (e.g., Foster et al. 2006; Hylander and Johnson, Hylander et al. 1987; Kay and Hiiemae 1974; Laird 2017). Further method development is needed to capture non‐vertical bite force.

In this synthesis, we compared methods used to estimate bite force in extant primates and provided a framework for bracketing and narrowing maximum bite force ranges. We advocate for moving beyond the view of bite force as a singular number in both extant and fossil primates, and instead recommend bracketing ranges for maximum bite force, utilizing multiple methods where possible. Even though the biological roles of bite force aren't always known or well understood for a given taxon, maximum bite force is routinely used as an indirect measure of fitness and a measure of craniodental performance for a variety of behaviors. Bracketing bite force between physiological and mechanical maxima should enable more meaningful comparisons between/among taxa and better inform modeling studies in both extant and fossil primates.

## Author Contributions

**Myra F. Laird:** conceptualization, investigation, funding acquisition, writing – original draft, methodology, validation, visualization, writing – review and editing, software, formal analysis, project administration, data curation, supervision, resources. **Megan A. Holmes:** writing – review and editing, methodology, project administration. **Claire E. Terhune:** writing – review and editing, methodology, project administration. **Andrea B. Taylor:** writing – review and editing, methodology, project administration.

## Conflicts of Interest

The authors declare no conflicts of interest.

## Data Availability

Data sharing not applicable to this article as no datasets were generated or analysed during the current study.

## Endnotes

^1^
Values were obtained using the same approach described in (Laird, Kanno, et al. 2023; Laird et al. 2024). Briefly, macaques were trained to bite on a force transducer for juice reward, and juice was occasionally withheld to encourage biting using higher force. Data collection was approved and collected under University of Pennsylvania IACUC protocol 805870.
